# Supplementary material for: Ultra-Small and Metabolizable Near-Infrared Au/Gd Nanoclusters for Targeted FL/MRI Imaging and Cancer Theranostics
Source: Biosensors (Basel). 2022 Jul 24;12(8):558. doi: 10.3390/bios12080558 (PMC9329954; doi:10.3390/bios12080558)
Supplement: Supplementary file 1 [file biosensors-12-00558-s001.zip › biosensors-1803996-supplementary.pdf]

Supporting Information

Ultra-Small and Metabolizable Near-Infrared Au/Gd Nanoclusters for Targeted FL/MRI Imaging and Cancer Theranostics

Xiawei Dong [1,†], Jing Ye [1,†], Yihan Wang [1], Hongjie Xiong [1], Hui Jiang [1], Hongbing Lu [2], Xiaohui Liu [1,*] and Xuemei Wang [1,*]

1 State Key Laboratory of Bioelectronics (Chien-Shiung Wu Laboratory), School of Biological Science and Medical Engineering, Southeast University, Nanjing 210096, China; dongxwseu@163.com (X.D.); jing832808@163.com (J.Y.); yihanwangxynu@163.com (Y.W.); 18056356092@163.com (H.X.); 101010998@seu.edu.cn (H.J.)
2 Department of Biomedical Engineering/Computer Application, Fourth Military Medical University,
Xi'an 710032, China; luhb@fmmu.edu.cn
* Correspondence: 101013182@seu.edu.cn (X.L.); xuewang@seu.edu.cn (X.W.); Tel.: +86-25-83792177 (X.L. & X.W.)
† These authors contributed equally to this work.

Supplemental Figures and Tables

Table S1 Maximum emission wavelength and fluorescence quantum yield of Au/Gd@BSA NCs with different mole ratios.

Number	molar ratio	Maximum emission wavelength (nm)	fluorescence quantum yield (%)
a	1:1	660	3.8%
b	1:1.5	690	4.4%
c	1:2	670	4.1%

Table S2 EDS of Au/Gd@FA NCs.

**Element**	**wt%**	**At%**
C	45.64	52.11
N	22.73	22.26
O	29.74	25.49
Au	1.72	0.12
Gd	0.16	0.02

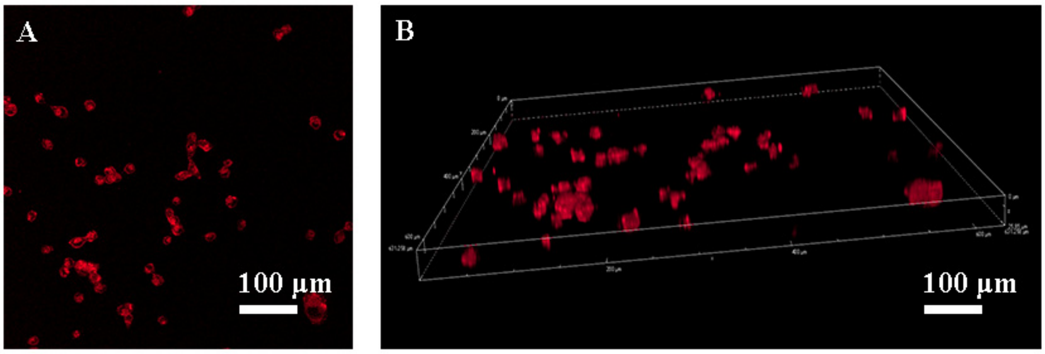

Figure S1. 3D confocal near-infrared fluorescence images of 4T1 cells after 6 h treatment with Au/Gd@FA NCs (60 µg/mL) at 37°C. (A) confocal near-infrared fluorescence images. (B) 3D confocal near-infrared fluorescence images.

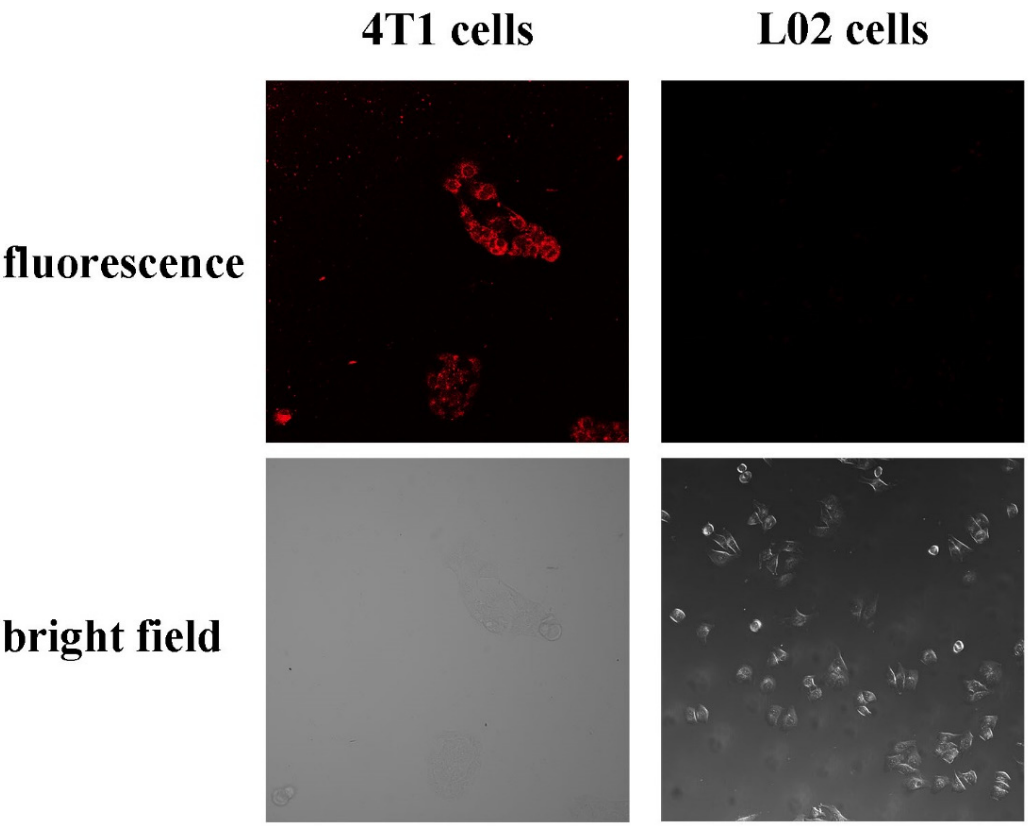

Figure S2. Confocal near-infrared fluorescence images of 4T1 cells and L02 cells after 6 h treatment with Au/Gd@FA NCs (60 μg/mL) at 37°C.

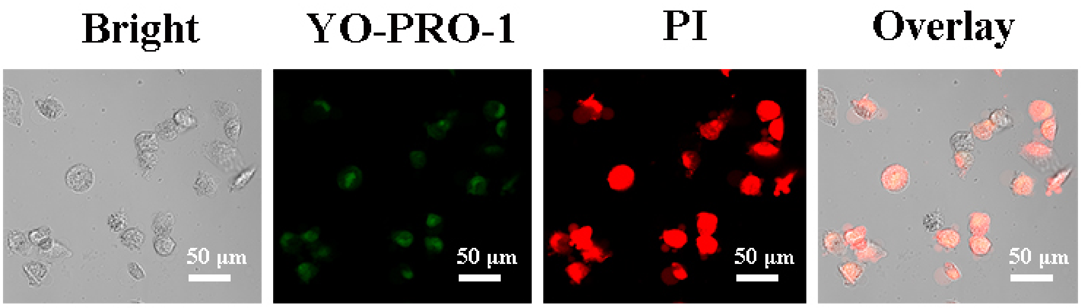

Figure S3. Confocal fluorescence images of 4T1 cells treatment with Au/Gd@FA NCs (60 μg/mL) at 37°C for 8h under 660 nm (1.2 W/cm^2) laser irradiation, which detected by YO-PRO-1/PI apoptosis kit.
